# Coblation Versus Bipolar Diathermy Hemostasis in Pediatric Tonsillectomy Patients: Systematic Review and Meta-Analysis

**DOI:** 10.7759/cureus.23066

**Published:** 2022-03-11

**Authors:** Mohammad Karam, Ahmad Abul, Abdulwahab Althuwaini, Abdulredha Almuhanna, Talal Alenezi, Ali Aljadi, Abdulrahman Al-Naseem, Abdulmalik Alsaif, Athari Alwael

**Affiliations:** 1 Medicine, Farwaniya Hospital, Kuwait City, KWT; 2 School of Medicine, University of Leeds, Leeds, GBR; 3 School of Medical Sciences, University of Manchester, Manchester, GBR; 4 Medicine, Walsall Healthcare NHS Trust, Birmingham, GBR; 5 Otolaryngology, Al Jahra Hospital, Al Jahra, KWT

**Keywords:** hemorrhage, post-operative pain, bipolar diathermy, coblation, tonsillectomy

## Abstract

This study aimed to compare the outcomes of coblation versus bipolar diathermy in pediatric patients undergoing tonsillectomy. A systematic review and meta-analysis were performed per the Preferred Reporting Items for Systematic Reviews and Meta-analyses (PRISMA) Guidelines. An electronic search of information was conducted to identify all Randomized Controlled Trials (RCTs) comparing the outcomes of coblation versus bipolar in pediatric patients undergoing tonsillectomy. Primary outcome measures were intraoperative bleeding, reactionary hemorrhage, delayed hemorrhage, and post-operative pain. Secondary outcome measures included a return to a normal diet, effects on the tonsillar bed, operation time, and administration of analgesia. Fixed and random-effects models were used for the analysis. Seven studies enrolling 1328 patients were identified. There was a significant difference between coblation and bipolar groups in terms of delayed hemorrhage (Odds Ratio [OR] = 0.27, P = 0.005) and post-operative pain (standardized mean difference [MD] = -2.13, P = 0.0007). Intraoperative bleeding (MD = -43.26, P = 0.11) and reactionary hemorrhage did not show any significant difference. The coblation group improved analgesia administration, diet and tonsillar tissue recovery, and thermal damage for secondary outcomes. No significant difference was reported in terms of operation time. In conclusion, coblation is comparable to a bipolar technique for pediatric patients undergoing tonsillectomy. It improves postoperative pain and delayed hemorrhage and does not worsen intraoperative bleeding and reactionary hemorrhage.

## Introduction and background

Tonsillectomy is one of the oldest surgical operations in medicine and is one of the most common operations of otolaryngologists [1,2]. Some potential indications for pediatric tonsillectomy include recurrent tonsillitis, sleep apnea, and PFAPA Syndrome (Periodic Fever, Aphthous Stomatitis, Pharyngitis, Adenitis), one of the most common operations during childhood [3]. Several techniques are used to perform tonsillectomies, including blunt dissection, guillotine, bipolar diathermy dissection, laser dissection, and the more recent coblation method [4]. Despite the range of available techniques, post-operative pain, primary or reactionary hemorrhage, and post-operative infection associated with the hemorrhage continue to present as the main post-tonsillectomy complications [5]. Therefore, studies continue to debate the optimal tonsillectomy technique.

Coblation (cold ablation) is a relatively new tonsillectomy technique that has earned increased popularity due to the decreased post-operative pain and reduced intraoperative bleeding that comes with its use [6]. Coblation is operated at surface temperatures (40-70°C) much lower than those of more traditional techniques [7]. Instead of relying on heat, coblation applies radiofrequency energy to a conductive natural salt solution, forming a plasma membrane comprised of highly ionized particles that hold enough energy to break the molecular bonds holding the tissue, thereby safely removing the target tissue [7-9]. The use of coblation eliminates the risk of causing thermal damage that comes with heat and minimizes necrosis of surrounding healthy tissue, therefore resulting in minimal pain and faster recovery [8,9].

Bipolar diathermy is an electrosurgery technique that functions by passing an alternating current at a high frequency through a pair of forceps to cut the tissue and coagulate the blood vessels [10]. Compared to monopolar diathermy, bipolar diathermy provides more control over the targeted area and uses less energy, thus causing less damage [10]. The use of bipolar diathermy to perform tonsillectomy was first described in 1994 by Pang et al. [11]. Bipolar diathermy tonsillectomy is proven to be safe for both adults and children [11,12]. Although the pain and morbidity rates are similar to other tonsillectomy techniques like cold dissection tonsillectomy, the bipolar diathermy technique provides significant advantages such as shorter operative time and lower blood loss levels [11,12].

Several published studies assessed the effectiveness of coblation compared with bipolar diathermy techniques in pediatric patients undergoing tonsillectomy [13-19], including a previous meta-analysis comparing coblation to several techniques used in tonsillectomy [20]. However, this is one of the few meta-analysis studies on these two specific techniques focusing on the pediatric population.

This article was previously posted to the medRxiv preprint server on September 14, 2020 (https://www.medrxiv.org/content/10.1101/2020.09.13.20193557v1).

## Review

Methods

A systematic review and meta-analysis were conducted per the Preferred Reporting Items for Systematic Reviews and Meta-Analyses (PRISMA) guidelines [21].

Eligibility Criteria

All randomized control trials and observational studies comparing coblation versus bipolar diathermy hemostasis techniques for tonsillectomy were included. Coblation was the intervention group of interest, and bipolar diathermy was the comparator. The study also included patients with bipolar scissors, bipolar forceps, cold steel, and bipolar diathermy hemostasis as the comparator. All patients were included irrespective of gender or co-morbidity status as long as they belonged to either a study or control group and were pediatric patients. All case reports cohort studies without a comparison group and studies not written in English were excluded. The study also excluded the following techniques: bipolar molecular resonance coagulation, tonsillotomy, unipolar or monopolar diathermy, adenotonsillectomy, and conventional tonsillectomy without explicitly stating bipolar diathermy as the primary method for hemostasis.

Primary Outcomes

The primary outcomes are intraoperative bleeding, reactionary hemorrhage (within 24 hours after the operation), delayed hemorrhage (bleeding after 24 hours), and post-operative pain on day 7.

Secondary Outcomes

The secondary outcomes included a return to a normal diet, effects on the tonsillar bed (degree of healing in tonsillar fossae and thermal damage to tonsillar tissue), operation time, and administration of analgesia.

Literature Search Strategy

Two authors independently searched the following electronic databases: MEDLINE, EMBASE, EMCARE, CINAHL, and the Cochrane Central Register of Controlled Trials (CENTRAL). The last search was run on December 5, 2021. Thesaurus headings, search operators, and limits in each of the above databases were adapted accordingly. In addition, World Health Organization International Clinical Trials Registry (http://apps. who.int/trial search/), ClinicalTrials.gov (http://clinical- trials.gov/), and ISRCTN Register (http://www.isrctn. com/) were searched for details of ongoing and unpublished studies. No language restrictions were applied in our search strategies. The search terminologies included "coblation", "bipolar", and "tonsillectomy". The bibliographic lists of relevant articles were also reviewed. 

Selection of Studies 

The title and abstract of articles identified from the literature searches were assessed independently by two authors. The full texts of relevant reports were retrieved, and articles that met the eligibility criteria of our review were selected. Any discrepancies in study selection were resolved by discussion between the authors.

*Data Extraction and Management* 

An electronic data extraction spreadsheet was created with Cochrane's data collection form for intervention reviews. The spreadsheet was pilot tested in randomly selected articles and adjusted accordingly. Our data extraction spreadsheet included study-related data (first author, year of publication, country of origin of the corresponding author, journal in which the study was published, study design, study size, clinical condition of the study participants, type of intervention, and comparison), baseline demographics of the included populations (age and gender), and primary and secondary outcome data. The authors cooperatively collected and recorded the results, and any disagreements were solved via discussion.

Data Synthesis

Data synthesis was conducted using the Review Manager 5.3 software. The extracted data were entered into Review Manager by three independent authors. The analysis involved used based on fixed and random effects modeling. The results were reported in forest plots with 95% Confidence Intervals (CIs).

For dichotomous outcomes, the Odds Ratio (OR) was calculated between the two groups. The OR is the odds of an event in the coblation group compared with the bipolar group. An OR of greater than 1 for the delayed hemorrhage would favor the coblation group, an OR of less than one would favor the bipolar group, and an OR of 1 would favor neither group.

The mean difference (MD) was calculated for continuous outcomes between the two groups. A positive MD for the post-operative pain score by day seven and intraoperative bleeding would favor the coblation group, a negative MD would favor the bipolar group, and an MD of 0 would favor neither group.

*Assessment of Heterogeneity* 

Heterogeneity among the studies was assessed using the Cochran Q test (χ2). Inconsistency was quantified by calculating I2 and interpreted using the following guide: 0% to 25% may represent low heterogeneity, 25% to 75% may represent moderate heterogeneity, and 75% to 100% may represent high heterogeneity.

Methodological Quality and Risk of Bias Assessment

Multiple authors independently assessed the methodological quality and the risk of bias for articles matching the inclusion criteria. Cochrane's tool for evaluating the risk of bias was used for randomized trials. Domains assessed included selection bias, performance bias, detection bias, attrition bias, reporting bias, and other possible sources of bias. Randomized controlled trial (RCT) studies are classified into low, unclear, or high risk of bias. For non-randomized studies, the Newcastle-Ottawa Scale was used [22]. It uses a star grading system to assess studies in three domains: selection, comparability, and exposure. The total maximum score for each study is nine stars. The overall rating of either good, fair, or poor quality was based on the Agency for Healthcare Research and Quality (AHRQ) standards [22].

Results

Literature Search Results

**Figure 1:**
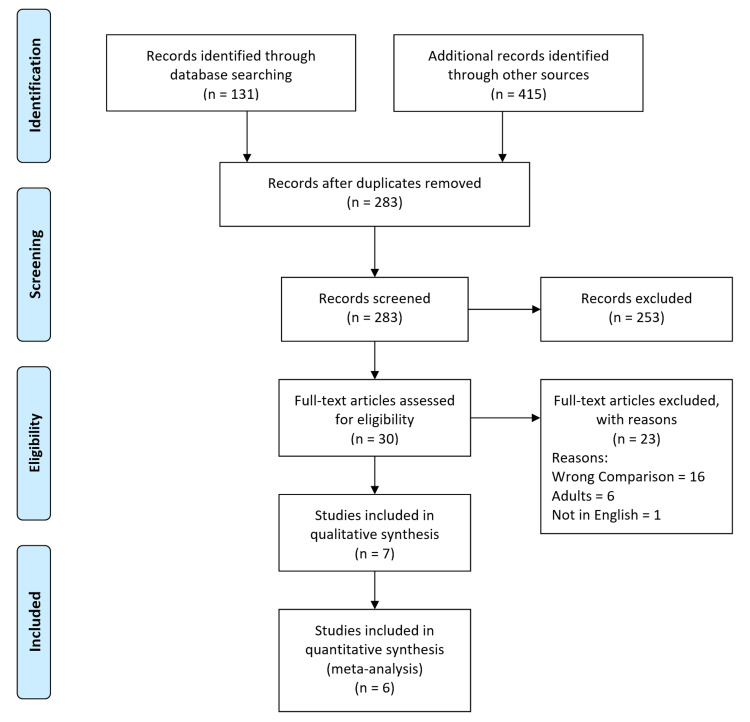
Prisma Flow Diagram. The PRISMA diagram details the search and selection processes applied during the overview. PRISMA: Preferred Reporting Items for Systematic Reviews and Meta-Analyses

The search strategy retrieved 546 studies. After a thorough screening of the retrieved articles using the inclusion and exclusion criteria, the authors identified seven studies in total that met the eligibility criteria (Figure 1).

Description of Studies

Table 1 summarises the baseline characteristics of included studies published from 2001 to 2018. All included populations were pediatric patients under 18 who underwent tonsillectomy.

Temple et al. [13] conducted a single-center prospective RCT that included 38 pediatric patients listed for a routine tonsillectomy with chronic tonsillitis or obstructive tonsils history. Patients were randomized via closed opaque envelope technique to either have bilateral coblation tonsillectomy (using an ArthroCare CoVac™ 70 ArthroWand®) or bilateral standard bipolar dissection tonsillectomy. All tonsillectomies were extracapsular.

Mitic et al. [14] performed a single-center prospective RCT that included 40 patients with standard indication for tonsillectomy. Randomizing was achieved by the closed opaque envelope technique to either have coblation tonsillectomy or dissection tonsillectomy with bipolar diathermy hemostasis. Bipolar dissection was done using standard technique (set at 4/10 and 50 Watts power). All tonsillectomies were extracapsular.

Parker et al. [15] performed a single-center prospective RCT that included 60 pediatric patients undergoing tonsillectomy by either cold steel dissection or coblator dissection. The trial was double-blinded, and a computer-generated random sequence in sealed opaque envelopes was used to allocate the procedure technique. All tonsillectomies were extracapsular.

Roje et al. [16] performed a single-center, prospective RCT that included 72 pediatric patients listed for tonsillectomy. Randomization was fulfilled by using computer-generated random numbers that separated the patients into one of two groups that would undergo either coblation tonsillectomy or conventional cold-steel tonsillectomy with bipolar diathermy. All tonsillectomies were extracapsular.

Belloso et al. [17] performed a single-center prospective observational cohort study which included 1008 participants from July 2001 to January 2003; 526 patients received a coblation tonsillectomy, and 482 patients received a blunt dissection tonsillectomy. Participants' data was extracted from a tonsillectomy audit. All tonsillectomies were extracapsular. Bipolar dissection was done using standard technique (set at 4/10- and 50-Watts power).

Konsulov et al. [18] carried out a single-center prospective cohort study which included 60 children aged 3-18 years. The children were divided into two equal groups. One group received traditional blunt dissection with bipolar diathermy hemostasis, and the other underwent coblation tonsillectomy using the ArthroCare CoVac™ 70 ArthroWand®. All tonsillectomies were extracapsular.

Bhardwaj et al. [19] conducted a single-center prospective RCT that included 100 pediatric patients undergoing tonsillectomy. Patients were given random numbers, and they got randomly allocated to one of two groups: one group undergoing bipolar diathermy and the other undergoing coblation. Bipolar dissection was done using 12 watts of power. All tonsillectomies were extracapsular.

**Table 1:** Baseline Characteristics of the Included Studies. NR: not reported. † NR: Not Reported ‡ RCT: Randomized Control Trial

Study (Year)	Country, Journal	Study Design	Sex (Male:Female)	Mean Age ± SD (Range)	Total Study Sample (Control:Intervention)	Interventions Compared
Temple et al. (2001) [13]	International Journal of Paediatric otorhinolaryngology, UK	RCT‡	19:19	5.6 years	38 (18:20)	Standard bipolar versus coblation tonsillectomy
Mitic et al. (2007) [14]	Clinical Otolaryngology, Norway	RCT	NR†	NR (4-12 years)	40 (20:20)	Coblator or steel dissecting instruments versus bipolar diathermy
Parker et al. (2009) [15]	Clinical Otolaryngology, UK	RCT	35:44	8.2 years (4-15 years)	70 (35:35)	Cold steel dissection with bipolar hemeostasis versus coblator dissection
Roje et al. (2009) [16]	Collegium antropologicum, Croatia	RCT	41:31	6 years (3-16 years)	72 (36:36)	Conventional cold steel tonsillectomy with bipolar diathermy coagulation versus coblator
Konsulov et al. (2017) [18]	International Journal of Otorhinolaryngology, Bulgaria	Prospective Comparative Study	NR	NR (3-18 years) Coblation: 8.16 ± 4.74 Bipolar: 6.87 ± 3.01	60 (30:30)	Coblator II system ArthroCare (Smith and Nephew) Evac 70 wand for extracapsular dissection vs. extracapsular blunt dissection with bipolar diathermy
Belloso et al. (2010) [17]	The Laryngoscope, UK	Cohort	NR	Coblation: 7.76 ± 3.56 years Bipolar: 7.60 ± 3.44 years	1008 (526:482)	Coblation or blunt dissection versus bipolar diathermy hemostasis
Bhardwaj et al. (2018) [19]	Indian Journal of Otolaryngology and Head & Neck Surgery, India	RCT	NR	NR (4-14 years)	100 (50:50)	Coblation Assissted verus Bipolar Diathermy

Primary Outcomes

Bleeding: Three bleeding outcomes were reported in the included studies, namely intraoperative hemorrhage, reactionary hemorrhage, and delayed hemorrhage.

**Figure 2:**
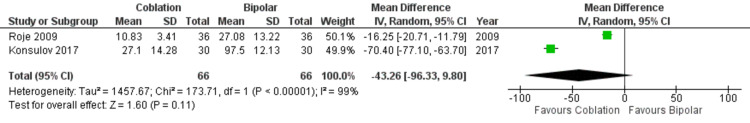
Forest Plot of Coblation versus Bipolar Tonsillectomy – Intraoperative Bleeding. Quantitative analysis showing the mean difference in the intraoperative bleeding reported as median by Roje et al. (2009) [16] and Konsulov et al. (2017) [18].

Two studies reported intraoperative bleeding included 132 patients, as shown in Figure 2. The mean difference analyses showed no statistically significant difference; however, a trend is demonstrated favoring the coblation group (MD = -43.26, CI = -96.33 to 9.80, P = 0.11). A high level of heterogeneity was found amongst the studies (I2 = 99%, P <0.00001). Mitic et al. [14] also reported less intraoperative bleeding in the coblation group (28.25 mL) than in the bipolar group (62.25 mL).

Reactionary or primary hemorrhage was reported by Roje et al. [16], who did not record any case of primary hemorrhage in both groups. However, Kunsulov et al. [18] reported two cases of reactionary hemorrhage in the bipolar group, compared to none in those that underwent coblation tonsillectomy.

**Figure 3:**
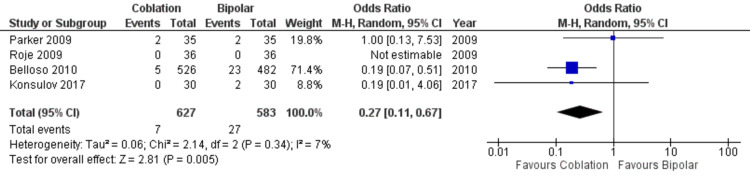
Forest Plot of Coblation versus Bipolar – Delayed Haemorrhage. Quantitative analysis showing the odds ratio in delayed haemorrhage reported by Parker et al. (2009) [15], Roje et al. (2009) [16], Belloso et al. (2010) [17] and Konsulov et al. (2017) [18].

Delayed or secondary hemorrhage was reported in four studies enrolling 1210 patients, as demonstrated in Figure 3. A statistically significant difference was seen in the odd ratio analyses showing a lower rate of delayed hemorrhage for the coblation group (OR = 0.27, CI = 0.11 to 0.67, P = 0.005). A low level of heterogeneity was found amongst the studies (I2 = 7%, P = 0.34).

**Figure 4:**
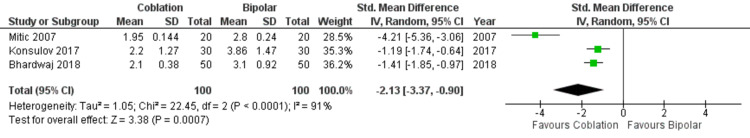
Forest Plot of Coblation versus Bipolar – Post-operative Pain by Day 7. Quantitative analysis showing the odd ratio in delayed haemorrhage reported by Mitic et al. (2007) [14], Konsulov et al. (2017) [18], and Bhardwaj et al. (2019) [19].

Post-operative pain by Day 7: In Figure 4, post-operative pain by day seven was reported using different pain scales in three studies enrolling 200 patients. A statistically significant difference was seen in the standardized mean difference analyses showing less pain for the coblation group (standardized MD = -2.13, CI = -3.37 to -0.90, P = 0.0007). A high level of heterogeneity was found amongst the studies (I2 = 91%, P < 0.0001). However, Temple et al. [13] also reported that mean post-operative pain scores did not include standard deviation; hence, it was not possible to quantitatively assess them in the forest plots. Temple et al. [13] reported a significant difference favoring coblation (p <0.0001).

Secondary Outcomes

Return to normal diet: Three studies with two different types of assessment of diet were included. Temple et al. [13] reported a statistically significant difference in the days it took to return to normal diet between the two groups, with an average of 2.4 days for patients who had coblation tonsillectomy versus an average of 7.6 days for patients who had routine bipolar dissection. On the other hand, Parker et al. [15] reported no significant difference in the number of days the two groups took to return to a normal diet, with a steady increase from day six onwards in both groups. Mitic et al. [14] reported diet using a nutrition score during a 10-day post-operative period. They found a statistically significant difference between patients who had coblation tonsillectomy scores and those who had dissection tonsillectomy across the ten days, favoring the former.

Effect on the tonsillar bed: A significant difference in the degree of healing in tonsillar fossae between the two interventions was reported by Temple et al. [13], with nearly all coblation fossae healed nine days post-operatively. At the same time, bipolar dissection patients had considerable slough. Roje et al. [16] reported a statistically significant mean difference of the depth of thermal damage (t = - 40,1; p<0.001) to tonsillar tissue, where the coblation technique caused damage two times shallower than that caused by bipolar diathermy hemostasis (428.58 ± 47.4 um and 841.17 ± 39.7 um, respectively).

Operation time (min): Operation time was defined as a point of a knife to skin contact according to Mitic et al. [14]. This study reported a statistically insignificant difference between coblation and bipolar groups (26.6 min and 25.6 min, respectively).

Administration of analgesia: Four studies have assessed analgesics administration in different ways. Parker et al. [15] identified fewer analgesic requirements by patients that underwent coblation tonsillectomy in the first 12 hours postoperatively. In addition, Roje et al. [16] and Konsulov et al. [18] agreed that patients undergoing coblation required a lower number of days on analgesics for the coblation group compared to those that received the bipolar technique. Roje et al. [16] also identified that the coblation group required fewer analgesic applications (4 vs. 8). Mitic et al. [14] has reported a lower medication intake score for the coblation group however did not specify how the scale works. Other than Roje et al. [16], the studies have mentioned using the same analgesics (ibuprofen and paracetamol).

Methodological quality and risk of bias assessment: The Cochrane Collaboration's tool was used to assess the quality of the RCTs included in the study (Table 2). The Newcastle-Ottawa scale was used to assess the quality of the non-randomized studies (Table 3) which offers a star system for analysis [22]. The quality of the included non-randomized study was deemed to be high in selection and exposure but low incomparability. Overall, both studies were of good quality based on the AHRQ standards [22].

**Table 2:** Bias analysis of the Randomized Trials using the Cochrane Collaboration’s Tool

First Author	Bias	Authors’ Judgement	Support for Judgement
Temple et al. (2001) [13]	Random sequence generation (selection bias)	Unclear Risk	No information given
	Allocation concealment (selection bias)	Low Risk	Closed opaque envelope technique
	Blinding of participants and personnel (performance bias)	High Risk	Single surgeon – not blinded.
	Blinding of outcome assessment (detection bias)	Low Risk	Outcome measurement is not likely to be influenced by lack of blinding
	Incomplete outcome data (attrition bias)	High Risk	Standard deviation for pain is not reported
	Selective reporting (reporting bias)	Unclear Risk	No information given
	Other bias	Low Risk	Similar baseline characteristics in both groups.
Mitic et al. (2007) [14]	Random sequence generation (selection bias)	Low Risk	A randomised sequence was generated by a statistician of the two interventions.
	Allocation concealment (selection bias)	Low Risk	Allocation of treatment was envelope sealed by a statistician and was then opened in the surgery room in sequential order by a nurse.
	Blinding of participants and personnel (performance bias)	High Risk	Surgeons were not blinded
	Blinding of outcome assessment (detection bias)	Low Risk	Outcome assessment was done by parents and nurses whom all were blinded for the operation method. The data from parents and nurses were compared and no difference was identified.
	Incomplete outcome data (attrition bias)	Low Risk	All questionnaires were complete.
	Selective reporting (reporting bias)	High Risk	Return to normal diet mentioned in abstract was not reported.
	Other bias	Low Risk	Similar baseline characteristics in both groups.
Parker et al. (2009) [15]	Random sequence generation (selection bias)	Low Risk	Random sequence generated by envelope
	Allocation concealment (selection bias)	Low Risk	Sealed opaque envelope technique
	Blinding of participants and personnel (performance bias)	High risk	Single surgeon.
	Blinding of outcome assessment (detection bias)	Low Risk	Double blinded study. The children, the parents and the nursing staff doing the pain assessments and prescribing the analgesia were not informed which technique had been used. The surgeon took no part in the pain assessments.
	Incomplete outcome data (attrition bias)	Low Risk	Missing outcome data balanced in numbers across intervention groups, with similar reasons for missing data across groups
	Selective reporting (reporting bias)	Low Risk	All outcome data reported
	Other bias	Low Risk	Similar baseline characteristics in both groups.
Roje et al. (2009) [16]	Random sequence generation (selection bias)	Low Risk	Computer-generated randomisation was used for selection of children and separating them into groups from a large ENT database.
	Allocation concealment (selection bias)	Unclear Risk	No information given
	Blinding of participants and personnel (performance bias)	High Risk	Single blinded (parents). Single surgeon not blinded
	Blinding of outcome assessment (detection bias)	Unclear Risk	Parents assessing secondary outcomes did not know which procedure was used.
	Incomplete outcome data (attrition bias)	High Risk	Participants lost to follow up (15 total- 7 coblation and 8 bipolar group)
	Selective reporting (reporting bias)	Unclear Risk	Insufficient information.
	Other bias	Low Risk	Similar baseline characteristics in both groups.
Bhardwaj et al. (2018) [19]	Random sequence generation (selection bias)	Unclear Risk	Did not explain the method of obtaining the random numbers
	Allocation concealment (selection bias)	Unclear Risk	Random numbers but not explicitly concealed
	Blinding of participants and personnel (performance bias)	Unclear Risk	Insufficient information
	Blinding of outcome assessment (detection bias)	Unclear Risk	Insufficient information
	Incomplete outcome data (attrition bias)	Low Risk	No missing information
	Selective reporting (reporting bias)	Low Risk	No published protocol but all the expected and pre-specified outcomes are reported
	Other bias	Low Risk	No significant demographic difference between the 2 groups.

**Table 3:** Newcastle-Ottawa Scale to Assess the Quality of Non-randomised Studies. *: stars to indicate points scored

Study	Selection	Comparability	Exposure
Belloso et al. (2010) [17]	****	*	***
Konsulov et al. (2017) [18]	****	*	**

Discussion

Coblation showed a superior effect when compared with bipolar diathermy in pediatric patients undergoing tonsillectomy in terms of delayed hemorrhage and post-operative pain, as shown by the results of the analyses. Although there were no significant differences between the coblation and bipolar groups regarding intraoperative bleeding (P = 0.11) and reactionary hemorrhage, delayed hemorrhage was significant (P = 0.005). Similarly, a significant (P = 0.0007) improvement in post-operative pain was noted for the coblation group. Regarding the between-study heterogeneity, it was low for delayed hemorrhage (I2 = 7%) and high for post-operative pain (I2 = 91%) and intraoperative bleeding (I2 = 99%), based on the assessment mentioned in the methodology.

Along with the outcomes mentioned above, the findings of this study reported several secondary outcomes that proved coblation to be a more effective technique than bipolar. Both tonsillar tissue recovery and thermal damage were significantly better in the coblation group. This correlates with analgesic administration whereby coblation required fewer doses than bipolar dissection. Generally, return to normal diet was quicker for the coblation group. There was no significant difference between the two groups regarding operation time.

Currently, there is a debate in the literature regarding the most efficient technique used for tonsillectomy in children [23]. The current study results are comparable to some studies where coblation tonsillectomy was found to be less painful than bipolar tonsillectomy in the immediate and overall post-operative period, which resulted in a swifter recovery and reduced analgesic requirements [24,25]. A randomized control trial that included 80 adolescents found that pain and otalgia post-operatively was slightly lower in the coblation group; however, this difference was considered clinically insignificant [25].

A more recent study in Iraq [26], which compared intraoperative efficiency and post-operative recovery between bipolar electrocautery and coblation tonsillectomy in children, recorded statistically and clinically significant higher amounts of intra-operative blood loss in bipolar technique than using coblation (Bipolar 1.43 ml vs. Coblation 15.37 ml, P <0.001). Although this outcome conflicts with the results of this study, the coblation technique was associated with lower mean pain scores, which is the direction of this study [26].

A study in the United States included 7,562 patients under 12 years of age compared the costs of treatment and management of children undergoing tonsillectomy by either coblation or electrocautery technique [27]. The study reported statistically significant lower costs for coblation compared to electrocautery surgery ($1,009 versus $1,162; P <0.0001) and pharmacy ($102.40 versus $117.20; P <0.0001) costs. However, when central supply is put into consideration the total cost of coblation is slightly higher [27] ($2,646 versus $2,591; P = 0.0011) with a difference of $55.94. However, because coblation is less likely to result in severe hemorrhage, it was found that using coblation tonsillectomy could save the National Health Service in the United Kingdom an incremental cost of £2000 for every avoided hemorrhage as opposed to using cold dissection with bipolar diathermy for hemostasis [28]. In a retrospective audit of 1,336 patients who had undergone coblation, it was reported that this technique had an increased requirement for operative intervention to manage secondary haemorrhage [29].

A systematic review and meta-analysis were performed on available studies in order to provide an evidence-based conclusion. The studies and their results were described and summarized, and the risk of bias in each study was appraised. The study designs and populations were standardized, with inclusion and exclusion criteria pre-defined. The interventions were homogenous across the included studies, with patients undergoing coblation tonsillectomy or bipolar diathermy. Although some variation was observed in the control group, such as using bipolar scissors as the primary cutting tool, bipolar diathermy was used as the primary technique for hemostasis. Despite these strengths, several inherent limitations should be considered. The current meta-analysis enrolled only seven studies with a total sample size of 1328 patients. There was a relatively large range in their sample sizes (1008 and 38 participants for Belloso et al. [17] and Temple et al. [13], respectively). Further clinical trials should therefore be completed to support the current study findings of the superior effect of coblation technique on pediatric patients undergoing tonsillectomy.

This study supports the coblation technique over bipolar diathermy for pediatric patients undergoing tonsillectomy. To further support the current conclusion, the authors suggest using coblation tonsillectomy under rigorous and well-designed RCTs to record data for primary and secondary outcomes appropriately.

## Conclusions

Although the evidence is limited, with only seven studies comparing coblation and bipolar techniques, the results of this meta-analysis suggest that coblation is a comparable option to a bipolar technique for pediatric patients undergoing tonsillectomy as it decreases delayed hemorrhage and post-operative pain and does not worsen intraoperative bleeding and reactionary hemorrhage. Coblation also has improved outcomes in the administration of analgesia, diet and tonsillar tissue recovery, and thermal damage. Further clinical studies are required to support the efficiency of coblation tonsillectomy technique.
